# Use of the Hyperbaric Chamber Versus Conventional Treatment for the Prevention of Amputation in Chronic Diabetic Foot and the Influence on Fitting and Rehabilitation: A Systematic Review

**DOI:** 10.1155/ijvm/8450783

**Published:** 2024-12-30

**Authors:** María Dolores Apolo-Arenas, Laura Guerrero-Nogales, César Luis Díaz-Muñoz, Berta Caro-Puértolas, José Alberto Parraca, Alejandro Caña-Pino

**Affiliations:** ^1^Department of Medical-Surgical Therapy, Medicine and Health Sciences Faculty, University of Extremadura, Badajoz, Spain; ^2^Research Group PhysioH (Fisioterapia e Hipoterapia), University of Extremadura, Badajoz, Spain; ^3^Department of Sport and Health, School of Health and Human Development, University of Évora, Évora, Portugal; ^4^Comprehensive Health Research Centre (CHRC), University of Évora, Évora, Portugal

**Keywords:** amputation, diabetic foot, hyperbaric oxygenation, rehabilitation

## Abstract

Diabetes mellitus (DM) is one of the most common chronic endocrine diseases, characterized by hyperglycemia, due to abnormal nitric oxide synthesis. The trend of an increase in the number of patients with DM continues. The medical and economic burden of DM is not only associated with hyperglycemia management but also with the management of DM-related complications. Most chronic DM–associated complications are vascular in nature. Thus, hyperbaric oxygen therapy (HBOT) can be used for primary and/or secondary prevention of vascular complications. This systematic review is aimed at providing an up-to-date analysis of the effects of HBOT in patients with diabetic foot ulcers (DFUs) on the prevention of amputation, fitting, and rehabilitation of amputees. The Preferred Reporting Items for Systematic Reviews and Meta-Analyses (PRISMA) guidelines were followed to conduct this systematic review. PubMed and Web of Science (WOS) database were employed in the search, which ended in November 2023. A risk of bias analysis was performed using the Evidence Project tool. After analyzing the records obtained, 10 studies were identified. However, seven fulfilled the inclusion criteria and were included in this systematic review. All included patients were over 18 years of age and had DM. The degree of DFU was assessed with the Wagner scale, being between 2 and 4, and the age of previous treatment of these DFU was taken into account. The results of the current systematic review showed that significant improvements can be achieved with HBOT when comparing its effects to those of the control group that followed usual care. Most studies included in the review showed positive results for DFU, amputation prevention, fitting, and rehabilitation of amputees. Therefore, the use of a hyperbaric chamber and standard care, as opposed to standard care alone, is favorable in patients with chronic DFUs. Promising and positive results were achieved for wound healing in DFU and the prevention of amputations.

## 1. Introduction

Diabetes mellitus (DM) is one of the most common chronic endocrine diseases in the 21st century and is characterized by chronically elevated blood glucose levels, known as hyperglycemia, which is caused by abnormal synthesis of nitric oxide (NO) [1, 2]. The trend of an increase in the number of patients with DM is continuing. The medical and economic burden of DM is not only associated with hyperglycemia management but also with the management of DM-related complications [3, 4].

In DM, we find a series of complications, among which the most common are vascular (pathophysiological changes in small blood vessels, which lead to the development of microangiopathy, tissue hypoxia, and ischemic lesions) [2, 5]. These complications are treated preventively, with the primary prevention being the management of hyperglycemia and the administration of treatments such as antiplatelet and lipid-lowering drugs. Alternatively, if these complications are already present, they can be secondary prevention, such as vascular protective management [2]. Impaired wound healing in patients with diabetes frequently leads to chronic leg and foot ulcers, which are serious complications. Diabetic foot ulcers (DFUs) are a complication of diabetes [6].

Currently, chronic diabetic foot is one of the most frequent complications of Type I or II diabetes. As a consequence of Type I or II diabetes, especially because of its influence on the appearance of DFU of different degrees, the worst case arrives at needing an amputation (either major or minor, depending on the amputation due to gangrene and necrosis) [5, 6].

The treatment usually carried out in DFU begins with optimal control of blood glucose levels. In addition, most patients with this type of ulcer also have underlying peripheral artery disease, requiring evaluation. The thick callus that forms around the DFU requires surgical debulking surgical overflow. Also, what is done is to offload the foot to remove pressure from the affected area, achieving this by means of certain shoes or other devices, but the most recommended is a full contact cast. And in other cases, they can benefit from the use of hyperbaric oxygen therapy (HBOT) [7].

HBOT could be used as both primary and secondary preventive vascular complication tool but also as an active treatment in some, such as diabetic foot [5]. HBOT is thought to assist wound healing, due to much increase in the dissolved oxygen in the plasma and tissue oxygen delivery [8]. Two other Cochrane systematic reviews showed that the potential value of HBOT for open fractures and burns was unclear [9, 10].

In this sense, one of the treatment options could be the hyperbaric chamber (HC), which is a type of treatment based on the provision of high partial pressures of oxygen by breathing pure oxygen inside the HC at a pressure higher than the atmospheric pressure. This objective is important because vascular complications associated with DM dramatically impact patients' quality of life and contribute to morbidity and mortality [11, 12].

This therapy in DM is used with certain parameters, which are the inhalation of 100% O_2_ under elevated atmospheric pressure of 1.6 to 2.8 ATA in HCs. This therapy is mainly used in ischemic conditions such as cerebral ischemia, peripheral artery disease, gangrenous wounds, and ischemia and reperfusion injury, as well as central retinal artery occlusion [5].

Currently, there are multiple options for the use of HCs, including hyperbaric medicine centers based on scientifically verified medical principles and hyperbaric medicine centers [13].

Therefore, this review is aimed at analyzing the effects and benefits of HBOT in patients with DFU in the prevention of amputations, fitting, and rehabilitation of amputees.

## 2. Methods

The present systematic review was performed according to the Preferred Reporting Items for Systematic Reviews and Meta-Analyses (PRISMA) guidelines [14] and following a similar methodology, a recent review published by our authorship [15], using the 27-item checklist, trying to follow the recommendations at each step of the process [16, 17]. The current study was registered in the international prospective register of systematic reviews of PROSPERO with the following identification number: CRD42023489520.

### 2.1. Data Sources and Search Strategy

PubMed and Web of Science databases (including Current Contents Connect, Derwent Innovations Index, Medline, and SciELO Citation Index) were used to identify potential studies. The MeSH descriptors were used: “Hyperbaric Oxygenation”; “Amputation”; “Dibetic Foot”; “Rehabilitation.”

Thus, the following search string was employed: (“Hyperbaric Oxygenation” AND “Amputation” AND “Diabetic foot”), (“Hyperbaric Oxygenation” AND “Amputation” AND “Rehabilitation”).

Studies were included in the systematic review if they fulfilled the following criteria: (1) clinical trials in English or Spanish; (2) published between 2008 and 2023; (3) studies on the use of the HC in chronic diabetic foot, with the aim of treating the ulcer and reducing the amputation rate, as well as the effects of the HC in the treatment of chronic diabetic foot, the objective of treating the ulcer and reducing the amputation rate, as well as the effects on prosthetic fitting; (4) studies with a control group; and (5) studies in which the patients received previous treatment without favorable results. Moreover, the studies were excluded when (1) they were written in a different language from English or Spanish; (2) they were a review, study protocol, conference abstract, or a case report; (3) they did not involve HC; (4) they do not include the influence on amputation rate; (5) they deal with lower limb injuries other than ulcers; and (5) they were animal studies.

The search process ended in November 2023. Duplicated studies were excluded, and articles' titles, abstracts, and full texts were carefully screened.

The study selection was performed by one author, M.D.A.-A., and checked by another, A.C.-P. In case of disagreement among the reviewers, it was resolved by consensus.

### 2.2. Risk of Bias Assessment

The Evidence Project tool [18] was employed to evaluate the risk of bias of the selected studies. This tool is composed of eight items that cover study design, the participants' representativeness, and the equivalence of comparison groups. In this regard, the study design includes items referred to cohort, control, or comparison group and pre–post-intervention data. Participants' representativeness includes items that analyze the random assignment of participants to the intervention, random selection of participants for assessment, and follow-up rate of 80% or more. Lastly, the comparison groups' equivalence is assessed with items concerning the equivalent on sociodemographics and the equivalent at baseline. This scale allows to evaluate both randomized and nonrandomized trials.

### 2.3. Data Extraction

According to PRISMA methodology [14], participants, intervention, comparison treatments, outcomes, and study design (PICOS) data were extracted. Accordingly, information concerning participants' characteristics, study design, sample size, age, severity of DFU according to the Wagner scale, and age of onset were exported from each article. Moreover, intervention characteristics such as intervention length, treatment frequency, duration of the sessions, and its description were analyzed. The extraction process was conducted by three authors (M.D.A.-A., L.G.-N., and A.C.-P).

**Figure 1:**
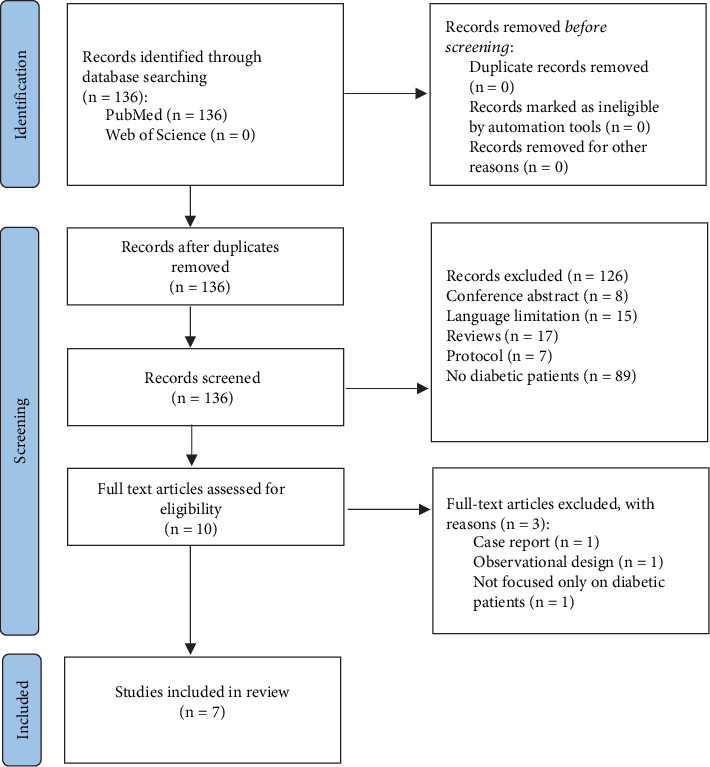
Flow diagram of the study selection.

Figure 1 shows the flowchart followed for the identification of relevant articles for the work, specifying those that were excluded for not being related to the objective of the work, for being duplicated, or for not having access to the full text of the article.

## 3. Results

### 3.1. Study Selection

A total of 136 publications were identified in the electronic databases: 136 studies in PubMed. One hundred and twenty-six studies were excluded because they were reviews (17 studies), conference abstracts (8 studies), and protocols (7 studies); had no diabetic patients (89 studies); or were not written in English or Spanish (15 studies). Ten studies were assessed for eligibility. However, three studies did not fulfil the inclusion criteria since one was a case report; the other excluded studies were an observational study and a study not focused only on diabetic patients. Therefore, our systematic review included seven studies (Figure 1).

### 3.2. Characteristics of the Participants

The results obtained are summarized in Table 1, which includes the authors of each study and their year of publication, in chronological order, study design, sample size, age, and types of participants in the control group.

Table 1 shows the study design, sample size, age, severity of DFU according to the Wagner scale, age of DFU, pretreatment and duration of DFU disability level, variables, and results for each article. Table 1 also shows the variables and results analyzed for each study. A total sample size of 545 participants was included in this systematic review. The largest sample size is 120 patients and the smallest is 30 patients. The mean age was 62.95. The mean age was 62.29 in the experimental group (EG) and 63.57 years in the control group (CG). All patients had Type I or Type II diabetes and were classified between II and IV according to the Wagner scale.

Information was collected on the age of the DFUs and whether they had been previously treated. All the studies included in the review indicate that all the patients have had previous treatment and the age of the DFU ranged from 1 to 6 months. In the case of Igor et al. [19], there are already unilaterally amputated patients in which the parameters analyzed in the other clinical trials are not assessable.

### 3.3. Objectives of the Studies

The main objective of these studies [6, 20–24] was to evaluate the efficacy of HBOT + SC (standard of care) in chronic DFU versus SC. In addition, Clarke and Hussey [20], Fedorko et al. [22], and Duzgun et al. [24] analyzed whether amputations in patients with diabetes decreased as well as improvements in wound healing.

In contrast, Igor et al. [19] evaluated the effects of HBOT + SPR (standard prosthetic rehabilitation) in patients with unilateral lower limb amputation.

### 3.4. Evaluated Measures

Different measures were assessed before and after intervention. All the studies included in this review [6, 19–24] evaluated sociodemographic characteristics of the patients: age, sex, duration of diabetes, arterial hypertension, body mass index, and glycosylated hemoglobin. In addition, all patients were classified according to DFU severity using the Wagner scale. In this regard, it was not applicable to Igor et al.'s study [19] and no results have been reported. The most studied outcome measures were the characteristics of the ulcers (location, size, infection, etc.) [6, 21, 22], the rate and need for amputation [21–24], ulcer healing [20, 22, 23], and the number of ulcers that healed [20, 22, 23]. Finally, the following variables were analyzed in a single study: markers of inflammation [21], quality of life [20], absence of major amputation [20], total or partial closure of DFUs without surgery [24], and mortality rate [23].

### 3.5. HC Parameters Used and Intervention Characteristics

The duration of HC intervention ranged from 2 to 8 weeks. Three interventions were performed for 2 and 4 weeks, while Clarke and Hussey [20] and Fedorko et al. [22] for 6 and 8 weeks. On the other hand, the number of sessions ranged from 20 to 40 sessions. The study that performed the fewest sessions was Chen et al. [21] with a total of 20 sessions, while Salama et al. [6], Clarke and Hussey [20], and Duzgun et al. [24] performed up to 40 sessions. Two studies did not indicate the frequency of sessions.

With regard to the characteristics of the HC sessions, all the studies included in the review used a single-place chamber, except for three studies [19, 20, 22], which used a multiplace chamber. Absolute atmospheric pressure ranged between 2.4 and 2.5 atm of pressure for 85–90 min with decompression periods of 5–15 min. The longest intervention was 120 min [22], while those of Igor et al.'s study [19] and Salama et al.'s study [6] were the shortest with a time of 60 min at 1.7 and 2.5 atm of pressure, respectively.

### 3.6. Effects of HC on the Variables Evaluated

Table 1 depicts the variables evaluated and the effects produced by HC therapy.

The application of HC in patients diagnosed with diabetes obtained positive effects on the healing and severity of DFUs [21, 23, 24], decreased amputation rate [16, 21, 24], and improved quality of life. In this sense, Chen et al. [21] obtained statistically significant results in terms of DFU severity (*p* = 0.010) and amputation rate (*p* < 0.05). On the other hand, Fedorko et al. [22] did not obtain significant differences between groups for amputation rate (*p* = 0.771) nor in DFU healing (*p* > 0.491). Clarke and Hussey [20] only obtained significant improvements with respect to the CE group in amputation rate, but there were no significant changes in complete DFU healing.

Thus, the study of Löndahl et al. [23] did determine the complete healing of patients treated with HC (*p* = 0.009 and *p* = 0.014) in the study of Salama et al. [6].

On the other hand, Duzgun et al. [24] obtained positive effects for the group that used CH, thus reducing the need for surgical interventions.

Regarding DFU surface area, there were significant changes at the end of treatment in the CH group in terms of DFU surface area (*p* = 0.001), while there were no significant changes in the conventional group (*p* = 0.126).

Finally, Igor' et al. [19] obtained a significantly higher increase in the percentage of arterial hemoglobin saturation in the group that received HC (*p* = 0.009). There were significant increase in pulse palpation frequency (*p* = 0.015), significant decrease in residual limb complications (*p* = 0.024) and residual limb strength (*p* = 0.000), and improvements in the Narang scale (P =0.038) and locomotor capacity index score (*p* = 0.048) in comparison with the control group. On the other hand, there were no significant changes for the increase in gait velocity and stride length over time on the 2-min gait scale (*p* = 0.081).

### 3.7. Risk of Bias

The mean score of the risk of bias analysis with the Evidence Project tool was 6 and scores ranged from 5 to 7 (Table 2). Higher scores corresponded to randomized controlled trial (RCT) studies (7/8) [21, 24] where assignment to experimental groups was randomized. Item by item analysis showed that assessment of the quality of the study design (Items 1, 2, and 3) was satisfactorily reached by all the studies. However, in the participants' representativeness evaluation, more heterogeneous results were found. Item 4, which assessed the “random assignment of participants to the intervention,” was fulfilled by all the studies, while Item 5 (“random selection of participants for assessment”) was not reached for any of the studies whereas only two studies [21, 24] positively scored Item 6 (“follow-up rate of 80% or more”). Besides, in the equivalence of comparison groups, except two studies in Item 7, all the studies fulfilled the requirements. Item 8 (which referred to the “comparison groups equivalent at baseline on outcome measures”) was satisfactorily reached by all the studies.

## 4. Discussion

The current systematic review analyzed the effects and benefits of HC in patients with DFU in the prevention of amputations, fitting, and rehabilitation of amputees. Seven articles were included in this systematic review: five were RCTs whereas two did not perform randomization. RCTs showed positive effects of HC on wound healing of DFU and prevention of amputations and in the rehabilitation of patients with diabetes included in the review. Furthermore, taking into account that non-RCTs studies are more prone to bias, the heterogeneity among the selected study results might be taken with caution.

Results of the current systematic review showed that significant improvements can be reached with HC when comparing its effects to CG that followed usual care. According to most of the studies included in the review, the DFUs of patients in the standard care group were treated by maintaining blood glucose, debridement of necrotic tissue, antibiotic therapy to treat infection, and wound care [6, 20–23]. Duzgun et al.'s study [24], in contrast to the other studies, although it includes the same treatment, also contains the main idea of resorting to the use of amputation when necessary, as a further treatment guideline.

Regarding the results related to the posttreatment healing rate of DFU, significant results were obtained by Chen et al. [21] (*p* < 0.05 in all the parameters measured for wound healing) and Salama et al. [6] (*p* = 0.014 in the HC group). In contrast to these results, in the trials by Fedorko et al. [22] and Clarke and Hussey [20], no significant changes in wound healing were obtained. Regarding the results of the variables posttreatment amputation rates (higher or lower), a considerable decrease of these amputations was obtained with *p* = 0.010 [20, 21, 24]. However, negative results were also obtained with respect to this parameter; according to Fedorko et al. [22], the decrease in amputations between the two groups was not significant with *p* = 0.771. On the other hand, Löndahl et al. [23] did not obtain significant changes either, since there were very similar results in terms of amputations in the HC group (3 major and 4 minor) and the CG (1 major and 4 minor). These results also coincide with Salama et al. [6], with no significant changes, since none of the groups had major amputations and only one minor amputation in both groups.

After analyzing the positive results in the most studied variables (wound healing of DFUs and amputation prevention) of the studies with the highest methodological quality [21, 24], we can conclude that Chen et al. [21] used 4 weeks for a total of 20 sessions with multiplace chamber (pressure of 2.5 ATA for 120 min). Duzgun et al. [24] used 20–30 days, where 2 sessions per day are applied with a monoplace chamber (pressure between 2 and 3 ATA for 90 min).

This systematic review had some limitations. First, only studies in Spanish and English were included. There is currently little literature on the use of HC for the treatment of DFUs to reduce amputations. Furthermore, the sample size of the studies included in the review was small. Second, some studies were not randomized, which could have affected the obtained results due to an increase of risk of bias in these studies. Therefore, RCTs with homogeneous populations are encouraged to assess the effect of HC in DFUs to ensure that the groups are equivalent at baseline. In addition, another future line of research could be to investigate the use of the HBOT in animals, for example, to treat diabetes-impaired wound healing in rats. Finally, after all that has been analyzed in this review, the use of HBOT points to a great utility in the prosthetic rehabilitation of lower limb amputees, although much research is still lacking.

## 5. Conclusion

The use of the HC and standard care, as opposed to standard care alone, is favorable in patients with chronic DFUs. Promising and positive results were achieved for wound healing of DFU and prevention of amputations.

## Figures and Tables

**Table 1:** Characteristics of the studies included in the systematic review.

**Study**	**Randomization**	**Group**	**Sample size**	**Age (SD)**	**Severity of DFU (I–V) according to the Wagner scale (** **n** **)**	**Age of DFU (SD)**	**Pretreatment/duration of DFU**	**Variables**	**Intervention**	**Results**
Salama et al. [6]	Non-RCT	EG: HBOT + SC	*n* = 15	55.1 (7.5)	I: 0 II: 13 III: 17 IV: 0 V: 0	≥ 3 months	Yes/at least 30 days	- Diabetes - DFU location - DFU surface measurements - Exudates - Type of tissue coverage - Infection - Major and minor amputation - Complete healing	2.5 atm/60 min at 100% 5 days/week 20–40 sessions	-DFU surface measurements: 50% (*p* = 0.0001) - Complete healing: 33.3% (*p* = 0.014) - Infection: 20% (*p* > 0.05) - Minor amputation: 6.5% (*p* > 0.05)
		CG: SC	*n* = 15	57.7 (6.7)		≥ 3 months	Yes/at least 30 days		N/A	-DFU surface measurements: 15% (*p* = 0.126) - Complete healing: 12% (*p* > 0.05) - Infection: 33.3% (*p* > 0.05) - Minor amputation: 6.5% (*p* > 0.05)

Clarke and Hussey [20]	Non-RCT	EG: HBOT + SC	*n* = 39	67.6	I: 0 II: 62 III: 36 IV: 22 V: 0	≥ 4 weeks	Yes/NR	- Major amputation - Freedom from any amputation - Complete healing - Recurrence of DFU - Quality of life - Mortality	2.4 or 2.5 atm/90 min at 100% 5 days/week 40 sessions	- Major amputation: 12% (*p* < 0.05) - Complete healing: 55% (*p* > 0.05) - Freedom from any amputation: 63% (*p* < 0.05) - Recurrence of DFU: 32% (*p* > 0.05)
		CG: SC	*n* = 81	70.6		≥ 4 weeks	Yes/NR		N/A	- Major amputation: 22% (*p* < 0.05) - Complete healing: 48% (*p* > 0.05) - Freedom from any amputation: 52% (*p* < 0.05) - Recurrence of DFU: 32% (*p* > 0.05)

Chen et al. [21]	RCT	EG: HBOT + SC	*n* = 20	64.3 (13.0)	I: 0 II: 15 III: 23 IV: 0 V: 0	59.1 (48.8) weeks	Yes/at least 1 month	- Inflammation index - Glycemic control - Blood flow - Quality of life from pretreatment, pretreatment to 2 weeks, and after completion of treatment - Hemoglobin A1c - Wound healing and survival rate of tissues, tissue survival rate - Amputation rate	2.5 atm/120 min 5 days/week for 4 weeks	- Complete DFU closure: 5 patients (25%) (*p* = 0.001) - Amputation rate: 5% (*p* = 0.010) - In the rest of the variables, no statistically significant differences were found (*p* > 0.05)
		CG: SC	*n* = 18	60.8 (7.2)		39.4 (33.6) weeks	Yes/at least 1 month		N/A	- Complete DFU closure: 1 patient (5.5%) (*p* = 0.001) - Amputation rate: 11% (*p* = 0.010) - In the rest of the variables, no statistically significant differences were found (*p* > 0.05)

Fedorko et al. [22]	RCT	EG: HBOT + SC	*n* = 49	61	I: 0 II: 46 III: 51 IV: 6 V: 0	≥ 4 weeks	Yes/NR	- Criteria for amputation (burden, persistent deep infection, pain) - Ulcer healing (size, wound assessment, and Wagner classification)	244 kPa/90 min 1 session/day 30 sessions	- Major amputation: 11 patients (*p* = 0.846) - Nonamputee recovered patients: 10 patients (*p* = 0.823) - Wound healing rates (*p* > 0.05)
		CG: SC	*n* = 54	62		≥ 4 weeks	Yes/NR		125 kPa/90 min 1 session/day 30 sessions	- Major amputation: 13 patients (*p* = 0.846) - Nonamputee recovered patients: 12 patients (*p* = 0.823) Wound healing rates (*p* > 0.05)

Igor et al. [19]	RCT	EG: HBOT + SPR	NR	61.2 (11.93)	NR	NR	NR	- Palpation of the pulse of the artery, dorsalis pedis in healthy lower limb - Pulse oximetry - Frequency of residual limb complications - Frequency of residual limb contractures - Circumference of the thigh and distal 1/3 of the leg of healthy lower limb - Muscle strength of the residual limb - Functional capacities: Narang scale, locomotor capacity index, and 2-min walking scale	1.7 atm/60 min at 100%, 15 sessions	Percentage of arterial hemoglobin saturation (*p* = 0.009) - Frequency of pulse palpation (*p* = 0.015) - Complications in the residual limb (*p* = 0.024) - Stump contractures (*p* = 0.731) - Residual limb circumference (*p* = 0.017) - Perimeter of the distal 1/3 of the leg (*p* = 0.374) - Muscular strength of the comparable residual limb (*p* = 0.000) - Narang scale (*p* = 0.038) - Locomotor capacity index score (*p* = 0.048) - Gait speed and stride length over time stride length over time (2-min walking scale) (*p* = 0.081) - Total standard prosthetic rehabilitation time and final discharge (*p* = 0.047)
		CG: SPR	NR	62.6 (11.52)		NR	NR		N/A	

Löndahl et al. [23]	RCT	EG: HBOT + SC	*n* = 48	69	I: 0 II: 51 III: 113 IV: 35 V: 0	≥ 3 months	Yes/at least 2 months	- Complete extremity healing - Ulcer healing - Major and minor amputation - Relapse - Death	2.5 atm/85 min 5 days/week 8 weeks/40 sessions	- Complete extremity healing: 52% (*p* = 0.03) - Ulcer healing: 61% (*p* = 0.009) - Major and minor amputation: 15% (*p* > 0.05) - Relapse: 19% (*p* > 0.05) - Death: 2% (*p* > 0.05)
		CG: SC	*n* = 42	68		≥ 3 months	Yes/at least 2 months		N/A	- Complete extremity healing: 29% (*p* = 0.03) - Ulcer healing: 27% (*p* = 0.009) - Major and minor amputation: 12% (*p* > 0.05) - Relapse: 19% (*p* > 0.05) - Death: 7% (*p* > 0.05)

Duzgun et al. [24]	RCT	EG: HBOT + SC	*n* = 50	58.1 (11.03)	I: 0 II: 18 III: 37 IV: 45 V: 0	≥ 4 weeks	Yes/NR	- Age - Gender - Duration of diabetes - Arterial hypertension - Lipoprotein lipid level - Obesity (body mass index) - Smoking - Glycosylated hemoglobin - Total healing without surgery - Closure with surgery - Distal amputation - Proximal amputation - No change in healing	Between 2 and 3 atm/90 min 2 sessions/day followed by 1 session/day From 20 to 30 days	- Nonsurgical healing: 0 patients (0%) - Closure with surgery: 8 patients (16%) - Distal amputation: 4 patients (8%) - Proximal amputation: 0 patients (0%) - No change in healing: 9 patients (18%) *p* < 0.05
		CG: SC	*n* = 50	63.3 (9.15)		≥ 4 weeks	Yes/NR		N/A	- Nonsurgical healing: 33 patients (66%) - Closure with surgery: 50 patients (100%) - Distal amputation: 24 patients (48%) - Proximal amputation: 17 patients (34%) - No change in healing: 0 patients (0%) *p* < 0.05

Abbreviations: CG, control group; EG, experimental group; HBOT, hyperbaric oxygen therapy; N/A, not applicable; NR, not reported; RCT, randomized controlled trial; SC, standard of care; SD, standard deviation; SPR, standard prosthetic rehabilitation.

**Table 2:** Risk of bias assessment.

**Study**	**Item 1**	**Item 2**	**Item 3**	**Item 4**	**Item 5**	**Item 6**	**Item 7**	**Item 8**	**Total score**
	**Study design**			**Participant representativeness**			**Equivalence of comparison groups**		
Salama et al. [6]	Yes	Yes	Yes	Yes	No	No	Yes	Yes	6/8
Clarke and Hussey [20]	Yes	Yes	Yes	Yes	No	No	Yes	Yes	6/8
Chen et al. [21]	Yes	Yes	Yes	Yes	No	Yes	Yes	Yes	7/8
Fedorko et al. [22]	Yes	Yes	Yes	Yes	No	No	No	Yes	5/8
Igor et al. [19]	Yes	Yes	Yes	Yes	No	No	Yes	Yes	6/8
Löndahl et al. [23]	Yes	Yes	Yes	Yes	No	No	No	Yes	5/8
Duzgun et al. [24]	Yes	Yes	Yes	Yes	No	Yes	Yes	Yes	7/8

*Note:* Item 1: cohort. Item 2: control or comparison group. Item 3: pre/postintervention data. Item 4: random assignment of participants to the intervention. Item 5: random selection of participants for assessment. Item 6: follow-up rate of 80% or more. Item 7: comparison groups equivalent on sociodemographics. Item 8: comparison groups equivalent at baseline on outcome measures.

## Data Availability

The authors have nothing to report.
